# Supplementary material for: PD-1 signaling uncovers a pathogenic subset of T cells in inflammatory arthritis
Source: Arthritis Res Ther. 2024 Jan 22;26:32. doi: 10.1186/s13075-023-03259-5 (PMC10801937; doi:10.1186/s13075-023-03259-5)
Supplement: Supplementary file 1 — Additional file 1. [file 13075_2023_3259_MOESM1_ESM.pdf]

Lot no.	Fluorochrome	Antigen	titrated?	optimal dilution	Antibody volume	buffer/total volume	Amount total	Comment
D0868083018133	UV450	GhostDye		1:10				
9262123	BUV395	ICOS			1	1		
9317667	BUV496	CD38			0.5	0.5		
0072072	BUV563	CD28		1:2	0.5	0.5		
0072070	BUV661	TCRdg			1	1		
0265647	BUV737	CCR6			1	1		
9305984	BUV805	CD8		1:2	0.5	0.5		
9009658	BV421	CD62L		1:10	0.1	1		
B269479	Pacific Blue	CD57		1:10	0.1	1		
0079161	BV480	GITR			1	1		
B288837	BV510	TIM-3			1	1		
B295384	BV570	CD45RA		1:10	0.1	1		
B249739	BV605	TIGIT			1	1		
B318075	BV650	LAG-3			1	1		
B239161	BV711	KLRG1			1	1		
0220284	BV750	CD5		1:30		1		
0072068	BV786	CD56			1	1		
7311760	BB515	CD27			1	1		
B290414	PerCP	CD69			1	1		
B267672	BB700	BTLA			1	1		
2134424	PerCP-eFlour710	CD185			1	1		
B220176	PE	CD161			1	1		
B289168	PE-Dazzle594	CD183			1	1		
B266014	PE-Cy5	CD95			1	1		
B200922	PE-Cy7	CD194			1	1		
B207596	APC	CD40L			1	1		
2127657A	APC-Cy5.5	CD4		1:2	0.5	0.5		
B239621	AF700	PD-1			1	1		
9172768	APC-Cy7	CD3		1:2	0.5	0.5		
B316395	APC-Fire810	HLA-DR		1:2	0.5	0.5		
	antibody volume					27ul		
	brilliant stain buffer volume					50ul		
	total volume					77ul		
