# Supplementary material for: PD-1 signaling uncovers a pathogenic subset of T cells in inflammatory arthritis
Source: Arthritis Res Ther. 2024 Jan 22;26:32. doi: 10.1186/s13075-023-03259-5 (PMC10801937; doi:10.1186/s13075-023-03259-5)

Supplement Figure 1

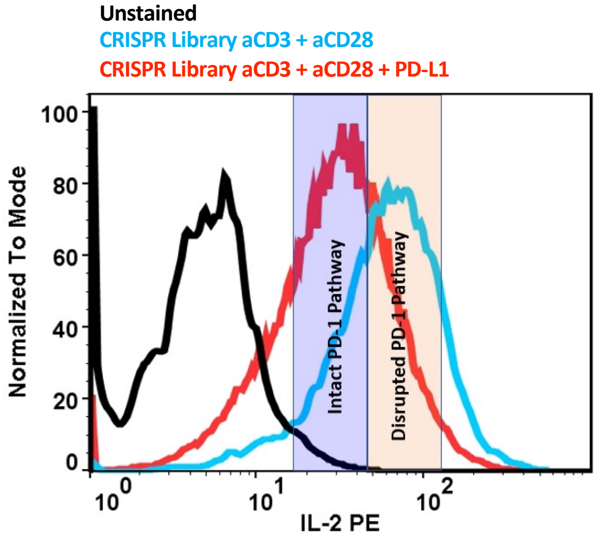

Supplement Figure 2

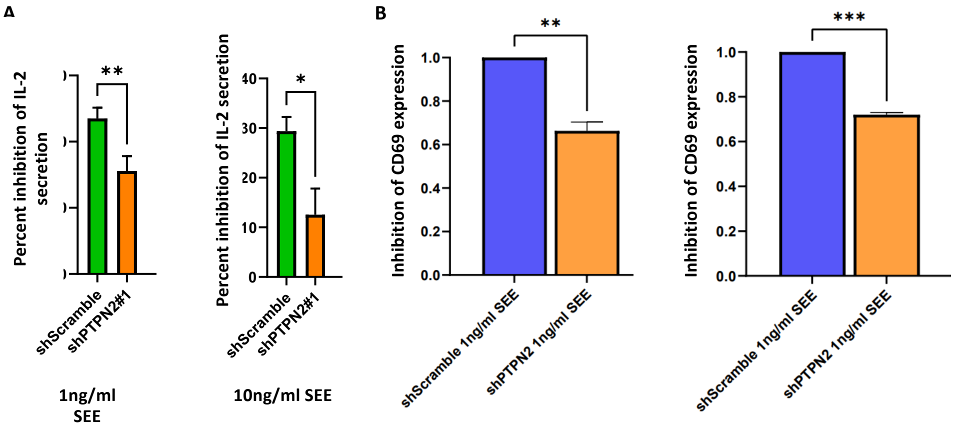

Supplement Figure 3

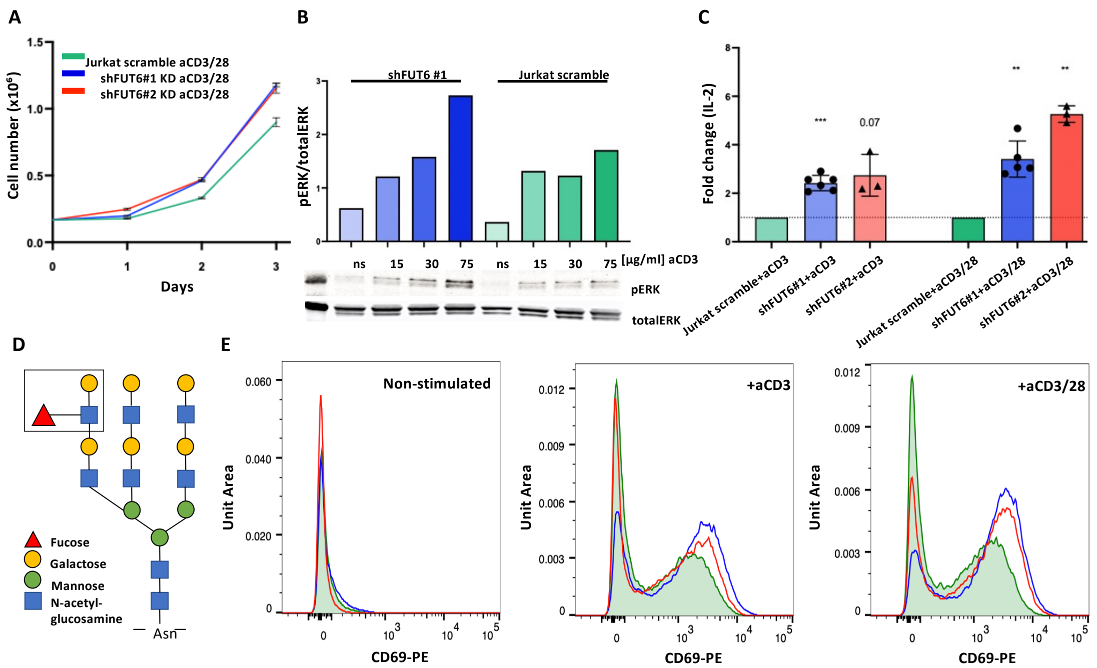

Supplement Figure 4

A

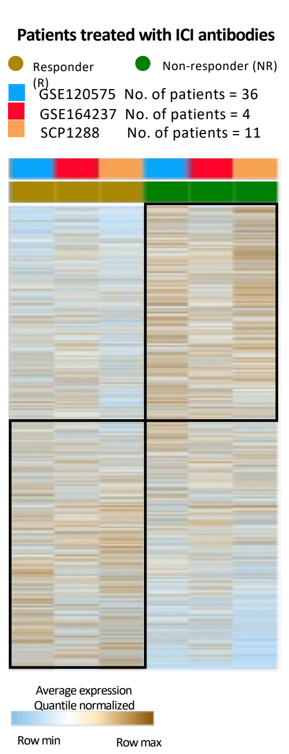

FTH1P18	NTS	SCGB1D2	CXCL6	PTH1R	KRT27	C20ORF78	NPR3	
RGS5	KIR2DL3	PDZK1	BAIAP2L2	RAD54L	CRYGC	APOL4	SLCO4C1	
C17ORF97	CST6	GSTA2	TNFRSF8	OLFML3	PTN	HMMR	VWA7	
PHACTR1	RNF43	SSTR1	CDC45	GPX6	KCND3	TRIO	PFN3	
KIF2C	PRLH	KRTAP6-2	NCF2	AP5B1	SPACA3	SULT2B1	ODAM	CRH
LYZL1	TEX11	ALDH8A1	TRAM1L1	BASP1	RHD	MEIS3	GRXCR2	
ASB14	ANLN	TSPAN1	TMEM217	CATSPER4	ROR1	GMPR	ZNF805	
SPDYE5	GLIPR1L1	GDA	BSX	CSF2RB	PNMA6A	DNMT3L	SDK1	
RIBC1	SMPDL3B	SUN5	KIR2DL1	KCNJ14	KANK4	ZNF667	BPIFB3	

PABPC5	SYT6	ZNF492	RND3	LYG1	PCDHGA1	SMAD9	SPRED3
STC2	SEMA5B	OR7G2	CTRL	CADM2	RAVER2	VCAN	OR6K2
CDH5	FCGR1A	JAK2	APC2	CSF2RA	C2ORF80	EVC2	FAM71A
NEUROD1	MTMR11	ACTA1	GJC1	CACNA2D2	TMEM47	PSD2	SLC9A4
GPR176	GPR179	NANOS3	PPP1R14C	IRX2	ACKR3	ASGR1	CARD9
TACR3	C17ORF78	CELF4	APCS	HORMAD1	TRPV6	NRCAM	FBN3
FST	TRPC5	CCKBR	FLT1	CXORF27	FAM229A	GPX2	GYPA
ZDHHC15	KCNE3	PNMT	HOXB5	NOXRED1	MAGEA2	OR10AD1	OR7D2
FNDC4	GSTT2	CLCN4	LTBP2	PLCD4	OVOL2	RIPK4	CDK18
DLC1	H1FNT	NOTCH3	SHISA9	PODN	TCL1B	KCTD1	FUT6
CHRNG	TCF21	P2RY1	C5ORF47	ABHD12B	MEOX2	S100A3	ACTL9
LAMP5	RBM46	CLLU1OS	CD300LG	UNC5C	PPFIA4	STMND1	LCE5A
KCNJ5	MIOX	FAM107A	CAMK2B	AP3B2	RFX4	HOXD1	

B

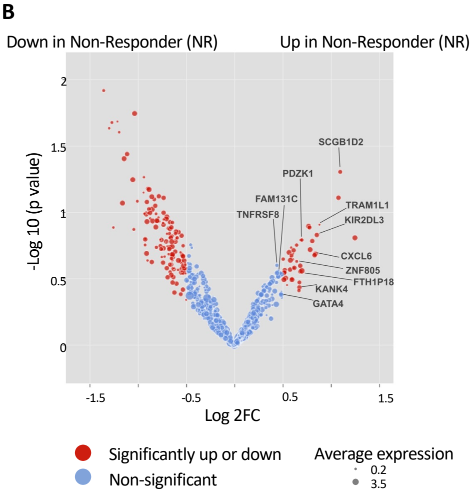

Supplement Figure 5

A

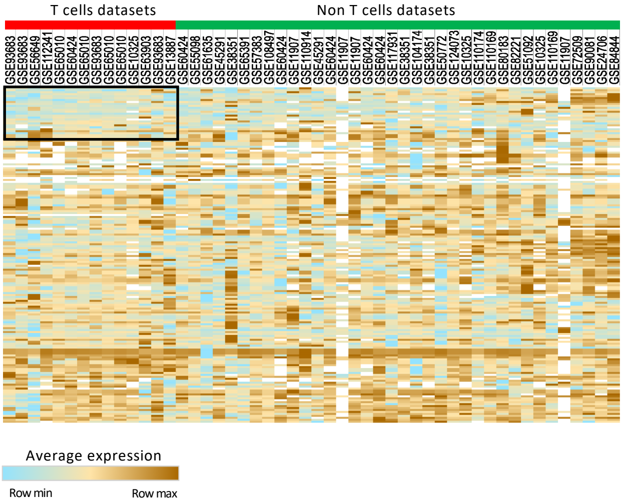

B

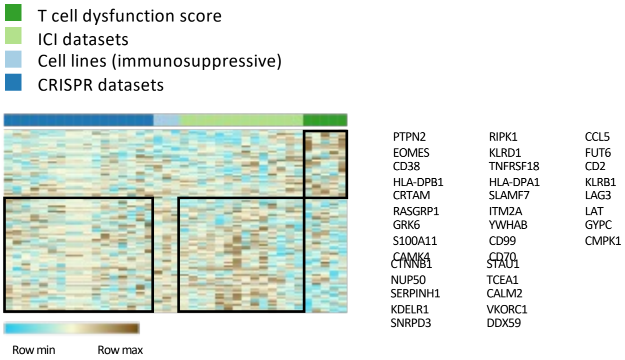

Ci

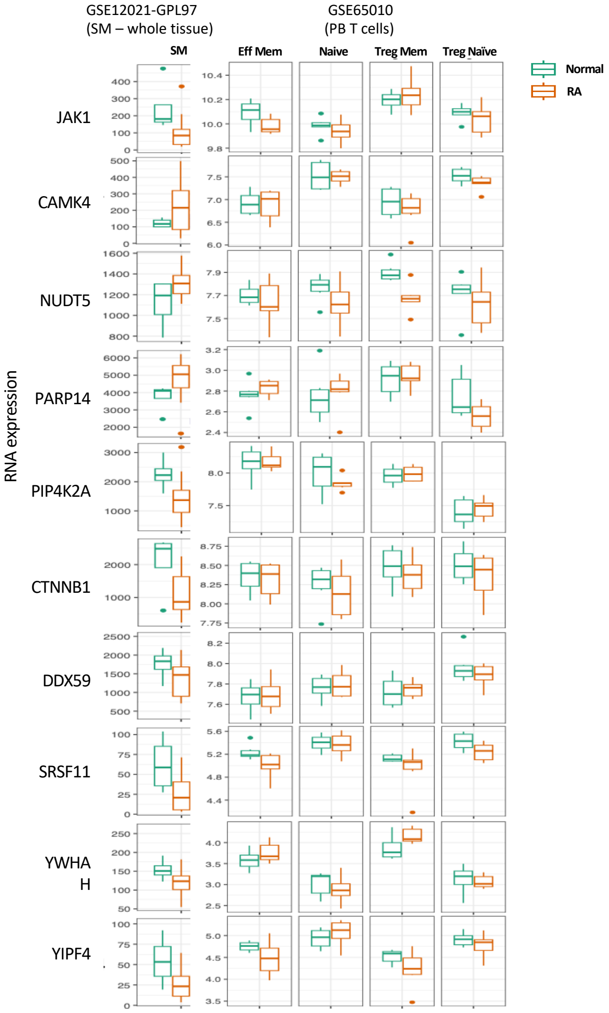

Cii

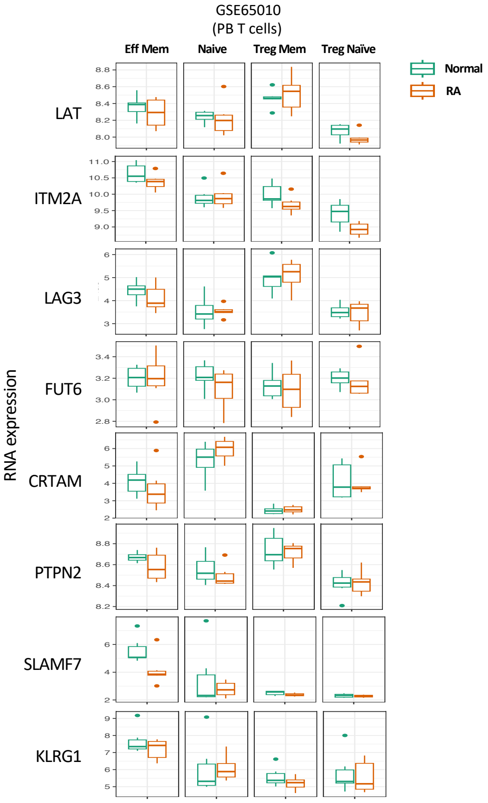

Supplement Figure 6

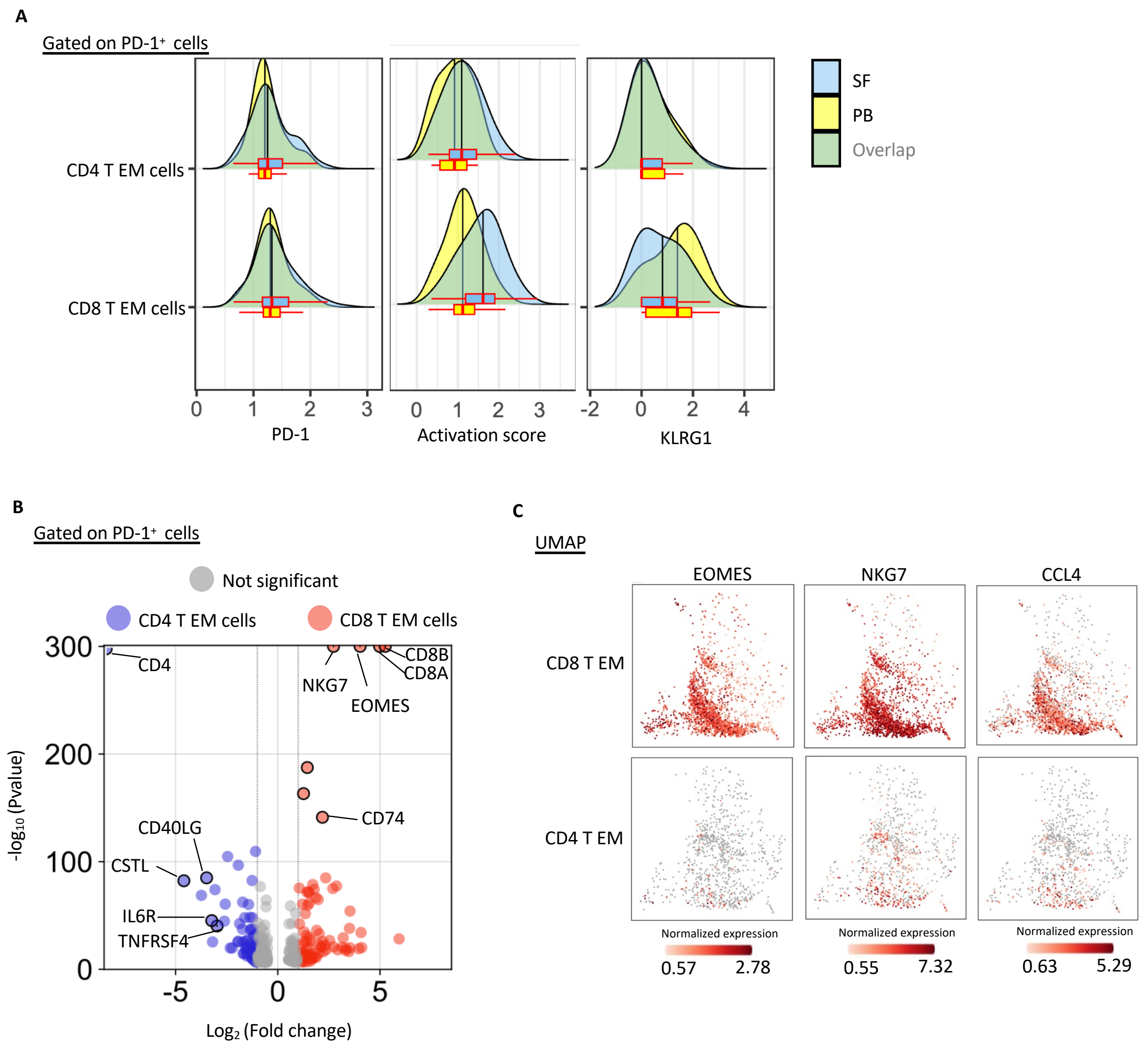

Supplement Figure 7

A

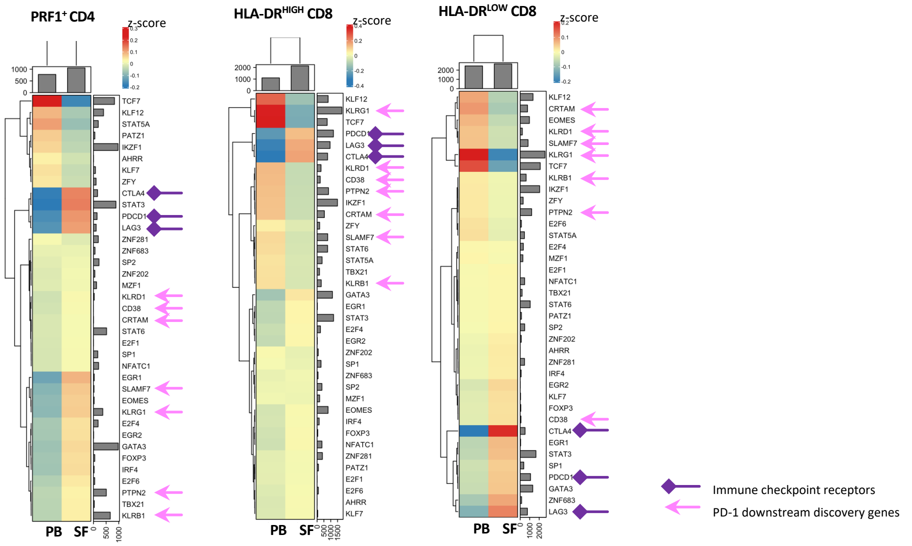

B

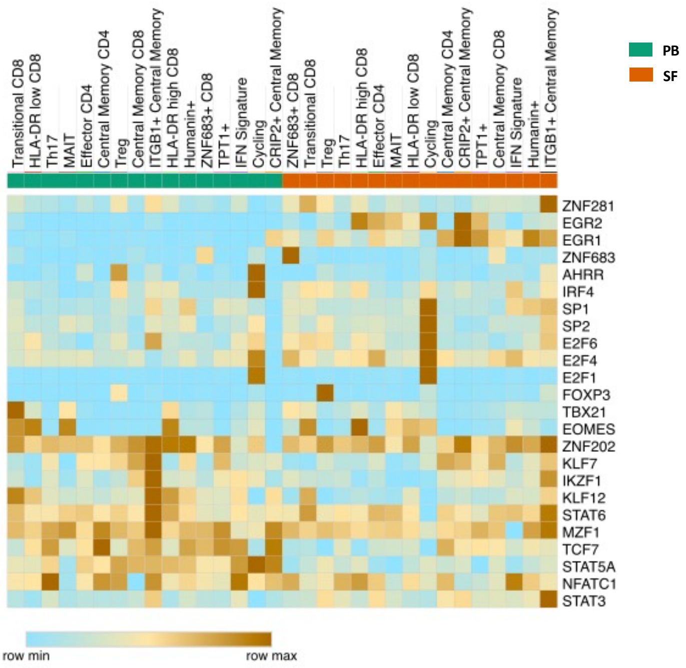

C

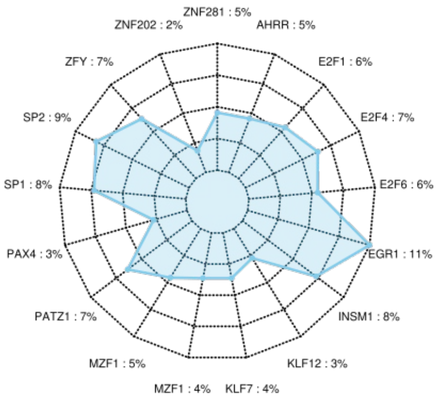

Supplement Figure 8

A

Gating strategy

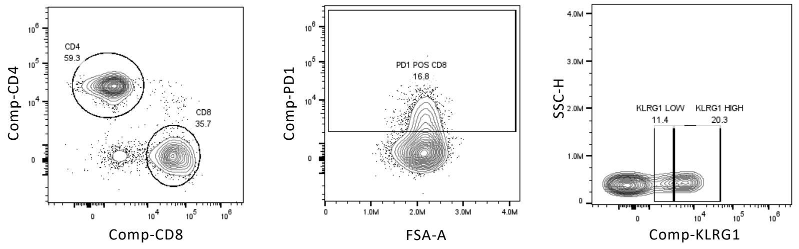

B

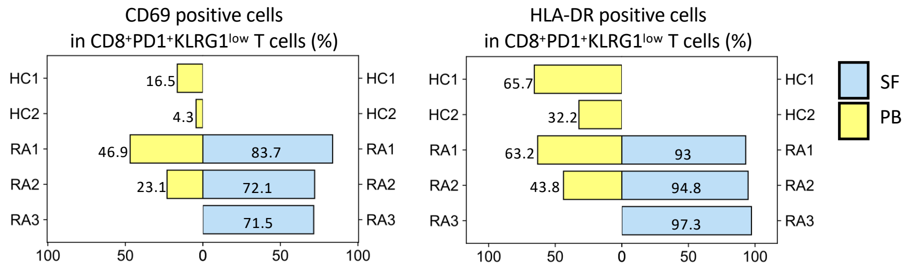

Supplement Figure 9

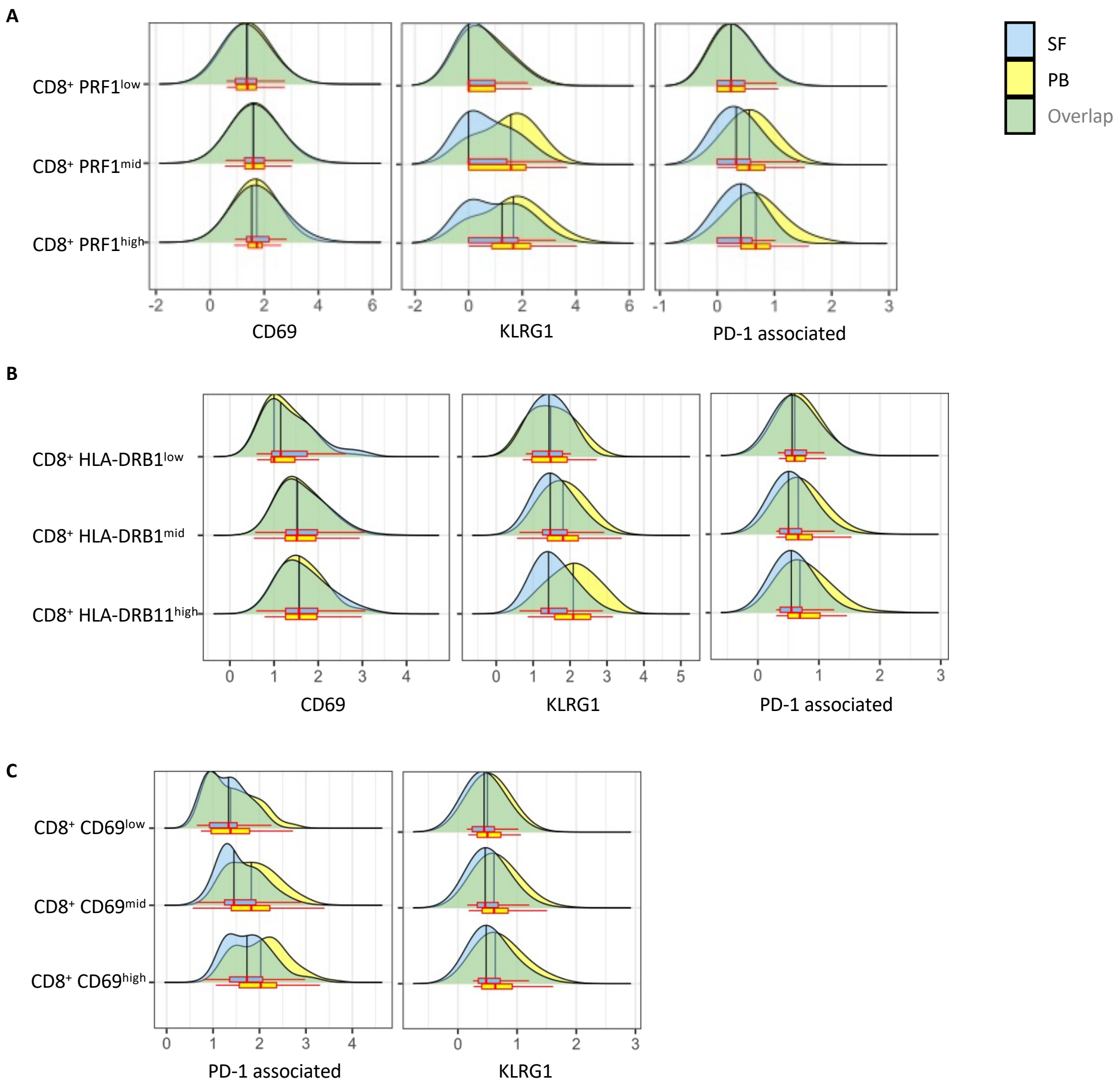

Supplement: Supplementary file 2 — Additional file 2: Supplement Figure 1. Flow cytometry histogram. A flow cytometry histogram shows the IL-2 expression levels of the cells sorted in the final stage of the Jurkat T cells CRISPR screen. Supplement Figure 2. PTPN2 is required for PD-1 functions. The percent of inhibition of secreted IL-2 from shirt hairpin (sh) scramble and sh PTPN2 Jurkat T cells occulted overnight with Raji B cell vs. Raji B cell that overexpressed PD-L1 at different concentrations of SEE (A). p<0.05, n=5. ELISA measured IL-2 levels. Flow cytometry quantifies CD69 expression inhibition from sh Scramble and sh PTPN2 Jurkat T cells occulted overnight with Raji B cell vs. Raji B cell that overexpressed PD-L1 in the presence of SEE (B). Two different shRPTN2 clones are shown at different concentrations of SEE (A). p<0.05, n=5. Supplement Figure 3. FUT6 signaling downstream of the T cell receptor. Growth assay of the different clones of short hairpin stably expressing Jurkat T cells stimulated with anti-CD3 and anti-CD28 over three days (A). Quantification of western blots showing the total ERK and phosphorylated ERK levels in the indicated sh Jurkat T cell lines stimulated with beads coated with antiCD3 for the appropriate time, as indicated in Figure (B). Fold change in secreted IL-2 levels from anti-CD3 stimulated shirt hairpin different Jurkat T cell lines measured by ELISA after overnight stimulation (C). n=3-5, *p<0.05. Cartoon showing the glycosylation pattern on FUT6 protein (D). Flow cytometry quantification of cell surface CD69 from Jurkat T cell line stably expressing the indicated sh and stimulated as indicated in Figure (E). Supplement Figure 4. The genes used to validate the dysfunctional T cell scores. Heatmaps showing differential expression levels of genes that we discovered to be involved in PD-1 downstream signaling and that are also differentially expressed in the other scRNA sequencing datasets (GSE120575, SCP1288, and GSE164237), as reported in Fig. 2D. The green co [file 13075_2023_3259_MOESM2_ESM.pdf]
